# Genetic Polymorphisms in Cytochrome P450 Enzymes Involved in Vitamin D Metabolism and the Vitamin D Receptor: Their Clinical Relevance

**DOI:** 10.3390/jpm15040128

**Published:** 2025-03-27

**Authors:** Yazun Jarrar, Ghayda’ Alhammadin, Su-Jun Lee

**Affiliations:** 1 Department of Basic Medical Sciences, Faculty of Medicine, Al-Balqa Applied University, Al-Salt 19117, Jordan; yazan.jarrar@bau.edu.jo; 2 Department of Pharmaceutical Science, College of Pharmacy, Al-Zaytoonah University of Jordan, Amman 11733, Jordan; 202027028@std-zuj.edu.jo; 3 Department of Pharmacology and Pharmacogenomics Research Center, Inje University College of Medicine, Inje University, Busan 50834, Republic of Korea

**Keywords:** vitamin D, vitamin D metabolism, genetic variants, vitamin D receptor, human diseases, single-nucleotide variants, cytochrome P450s

## Abstract

Individual variations in the active form of vitamin D (Vit.D) arise from a combination of dietary intake, sun exposure, and genetic factors, making it complex and challenging to maintain optimal levels. Among Vit.D-related genes, variations in *CYP2R1* and *CYP27B1* influence Vit.D synthesis, *CYP24A1* regulates its inactivation, and the Vit.D receptor (VDR) mediates Vit.D signaling. These genetic variations contribute to substantial differences in Vit.D concentrations and associated clinical effects. However, there has been a lack of comprehensive, simultaneous exploration of these key genes and their clinical implications. This review provides a systematic analysis of genetic variants in Vit.D-related P450 genes identified in human clinical studies, along with in silico predictions of their functional consequences. Since multiple genes seem to influence the body’s response to Vit.D, studying just one genetic variant may not fully explain Vit.D deficiency. A comprehensive evaluation of all Vit.D-related genes could offer valuable insights for advancing personalized medicine in Vit.D management. This study provides a foundation for developing a more personalized approach to Vit.D supplementation and regulation, guided by genetic information.

## 1. Introduction

Vitamin D (Vit.D) plays a critical role in calcium absorption and homeostasis and has been extensively studied in the context of bone density and skeletal health. However, emerging evidence has expanded its relevance to a broader range of physiological and pathological processes, including diabetes, hypertension, cardiovascular disease, autoimmune disorders, cancer, and depression [1]. Inter-individual variation in circulating 25-hydroxyVit.D (25-OH Vit.D) levels exceeds a 30-fold range [2], driven by multiple factors such as sun exposure, dietary intake, age, sex, pharmacological interactions, disease status, and genetic predisposition [3]. Among these, genetic factors contribute significantly to variability in 25-OH Vit.D levels, accounting for an estimated 23–83% of the observed differences [4,5]. Notably, genes involved in Vit.D metabolism, including those regulating its biosynthesis and clearance, have been identified as major determinants of inter-individual variation [6]. For instance, polymorphisms in *CYP27B1* and *CYP2R1* have been implicated in calcium-related disorders such as Vit.D-dependent rickets type 1 (VDDR1) [7,8,9]. Furthermore, genetic variants in *CYP2R1*, *CYP24A1*, and *Vit.D receptors (VDRs)* have been associated with altered circulating 25-OH Vit.D concentrations in a gene-dependent manner [4,10]. Specifically, mutations in *CYP24A1* have been linked to dysregulated Vit.D metabolism and hypercalcemia [11]. The biological impact of genetic variants is highly diverse, ranging from complete loss-of-function mutations that abolish enzymatic activity to neutral polymorphisms with no discernible effect on gene function or expression [12]. Functional consequences of genetic variants include amino acid substitutions leading to altered protein function, premature stop codons resulting in truncated proteins, splicing variants that modify mRNA length and stability, frame-shift mutations caused by nucleotide insertions or deletions leading to premature termination codons, and regulatory variants that influence gene expression levels [13]. A comprehensive understanding of the functional consequences of these genetic variants remains limited, making it challenging for clinicians and patients to accurately address Vit.D deficiency based on genotyping a limited set of variants, often relying on assumptions or statistical predictions. To ensure optimal Vit.D levels, it is more reliable and clinically beneficial to genotype for well-characterized functional variants or those previously validated in human studies. Importantly, Vit.D deficiency may result from non-genetic factors such as drug interactions, hepatic or renal impairment, or insufficient sun exposure, leading to potential misclassification as a genetic disorder [6]. Conversely, mutations in genes involved in Vit.D biosynthesis may obscure the phenotypic manifestation of other genetic variants associated with Vit.D deficiency, complicating accurate diagnosis due to overlapping environmental or clinical factors, as well as interactions with other Vit.D-related genetic variants. This uncertainty has led to reported hesitancy among both clinicians and patients regarding the clinical utility of genotyping in this context [14]. In this review, we examine the functional roles of Vit.D-related genetic variants that have been experimentally characterized. Furthermore, we summarize the genetic variants that exhibit strong associations with human diseases and emphasize the need for further investigation into uncharacterized variants through in silico analyses and functional validation studies.

## 2. Vitamin D

**Figure 1:**
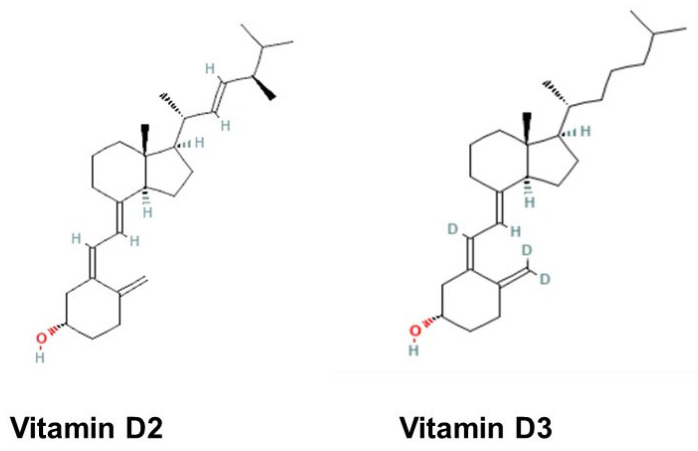
The structure of vitamin D2 and vitamin D3. The structures were obtained from the NCBI database (https://pubchem.ncbi.nlm.nih.gov/).

Vit.D is an essential fat-soluble secosteroid required for maintaining calcium and phosphate homeostasis, thereby ensuring proper bone mineralization, muscle function, and various cellular processes. First identified by McCollum et al. in 1922, Vit.D was hypothesized to facilitate calcium deposition, and subsequent studies demonstrated its role in preventing rickets, a metabolic disorder characterized by defective bone mineralization due to impaired calcium and phosphate metabolism [15]. It was later established that both sunlight exposure and dietary sources, such as cod liver oil, could prevent and treat rickets by increasing Vit.D levels [16]. Genetic mutations affecting Vit.D metabolism, as well as dietary deficiencies, have been identified as primary contributors to this disorder [17]. Vit.D exists in two primary forms: Vit.D3 (cholecalciferol), primarily derived from animal sources, and Vit.D2 (ergocalciferol), obtained from plant and fungal sources. In 1924, Steenbock and colleagues at the University of Wisconsin demonstrated that irradiating yeast enhanced its Vit.D2 content, leading to the fortification of milk as a strategy to prevent rickets [18]. Structurally, Vit.D is classified as a secosteroid, sharing a core four-ring backbone typical of steroidal compounds, with a characteristic broken ring structure (Figure 1). Both Vit.D2 and Vit.D3 are biologically inactive and require sequential hydroxylation, first at the 25th carbon in the liver and then at the 1st carbon in the kidneys, to produce the active hormone calcitriol (1,25-dihydroxyVit.D). Calcitriol plays a pivotal role in regulating calcium and phosphate metabolism, thereby contributing to skeletal integrity, immune function, and various physiological processes [19].

## 3. The Metabolism and Bioactivity of Vit.D

**Figure 2:**
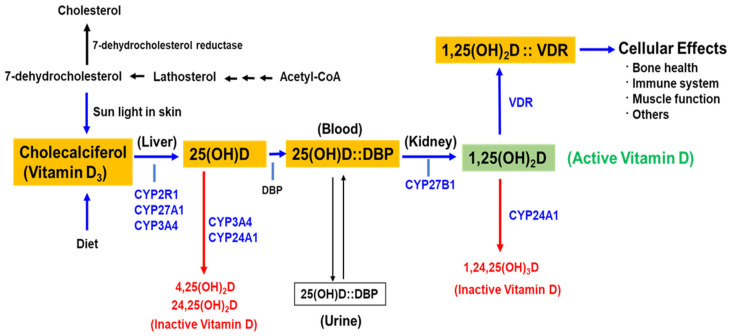
The metabolism and activation pathway of vitamin D. Vitamin D can be obtained from the diet or synthesized in the skin from 7-dehydrocholesterol upon UVB sunlight exposure. In the liver, vitamin D is converted to 25-hydroxyvitamin D [25(OH)D], the major circulating form, via CYP2R1. This is transported in the blood bound to vitamin D-binding protein (DBP) and further hydroxylated in the kidney by CYP27B1 to form 1,25-dihydroxyvitamin D [1,25(OH)2D], the active form. Active vitamin D binds to the vitamin D receptor (VDR) to regulate calcium and phosphate homeostasis and mediate various cellular effects. Excess vitamin D is inactivated by CYP24A1, which converts it into the metabolites 1,24,25(OH)3D for excretion.

Humans obtain Vit.D through two primary sources: endogenous synthesis of Vit.D3 in the skin upon exposure to ultraviolet B (UVB) radiation and exogenous intake from dietary sources, fortified foods, and supplements. Cutaneous synthesis accounts for approximately 90% of total Vit.D production, while dietary sources, including egg yolks, oily fish, shiitake mushrooms, organ meats, and liver, contribute to the remaining fraction [20]. Upon exposure to UVB radiation (wavelength 290–315 nm), the precursor molecule 7-dehydrocholesterol (7-DHC), present in the epidermal cells, undergoes photochemical conversion to pre-Vit.D3. Subsequent thermal isomerization results in the formation of Vit.D3. Similarly, Vit.D2 is derived from plant and fungal sources through a comparable process. However, both Vit.D3 and Vit.D2 are biologically inactive and require enzymatic activation through sequential hydroxylation. As illustrated in Figure 2, the first hydroxylation occurs in the liver, where the enzyme CYP2R1 catalyzes the conversion of Vit.D into 25-hydroxyVit.D [25(OH)D], also referred to as calcidiol. This metabolite, the primary circulating form of Vit.D, has a half-life of approximately two weeks and serves as a biomarker for assessing Vit.D status [21]. The second hydroxylation step occurs predominantly in the kidneys, where the enzyme CYP27B1 catalyzes the conversion of 25(OH)D into its biologically active form, 1,25-dihydroxyVit.D [1,25(OH)2D], also known as calcitriol. This conversion is tightly regulated by parathyroid hormone (PTH) in response to serum calcium and phosphate levels, as well as other mediators such as growth hormone (GH) [22]. Beyond renal hydroxylation, extra-renal tissues—including keratinocytes, osteoblasts, lymph nodes, placenta, colon, and alveolar macrophages—express CYP27B1, enabling local conversion of 25(OH)D into 1,25(OH)2D. This suggests an autocrine–paracrine role for calcitriol in various physiological processes [21]. In murine models, CYP2R1 knockout results in a significant (>50%) reduction in circulating 25(OH)D levels, although complete depletion does not occur, indicating the presence of alternative metabolic pathways. In humans, mutations in CYP2R1 and CYP27B1 have been implicated in hereditary rickets, further underscoring their critical role in Vit.D metabolism [23]. Additionally, CYP3A4, an enzyme primarily involved in xenobiotic metabolism, contributes to Vit.D catabolism by hydroxylating calcitriol, thereby reducing its biological activity and facilitating its degradation. This enzymatic regulation plays a crucial role in maintaining the balance between active and inactive forms of Vit.D, particularly in extra-renal tissues [24]. Furthermore, CYP27A1, a mitochondrial enzyme predominantly expressed in the liver, hydroxylates 25(OH)D at the 24-position, leading to the formation of 24,25-dihydroxyVit.D, an inactive metabolite. This catabolic pathway serves as a protective mechanism against Vit.D toxicity and plays a key role in calcium homeostasis [25].

## 4. Vit.D Receptor

Vit.D-binding protein (VDBP), a member of the albumin superfamily, plays a crucial role in the transport and distribution of Vit.D metabolites. Approximately 85% of circulating 25-hydroxyVit.D [25(OH)D] and 1,25-dihydroxyVit.D [1,25(OH)2D] are bound to VDBP, facilitating their delivery to target tissues. However, studies have shown that the absence of VDBP does not necessarily result in Vit.D deficiency unless dietary intake is severely restricted, suggesting the presence of compensatory mechanisms for Vit.D homeostasis [26]. The biological actions of Vit.D are mediated through the activation of the cytosolic Vit.D receptor (VDR), a ligand-dependent transcription factor belonging to the nuclear receptor superfamily. Acting as a hormone, 1,25(OH)2D binds to VDR and translocates into the nucleus of target cells, where it regulates gene transcription. The ubiquitous expression of VDR across various tissues underscores the extensive physiological functions of Vit.D. It is estimated that 1,25(OH)2D directly or indirectly modulates the expression of approximately 1250 genes by binding to VDR and interacting with Vit.D response elements (VDREs) in promoter regions, thereby activating or repressing transcription [27]. Structurally, VDR shares common features with other nuclear receptors, including a DNA-binding domain, a ligand-binding domain, a highly conserved N-terminal domain of 23 amino acids, and a flexible hinge region. Upon activation, VDR typically forms a heterodimer with one of the three retinoid X receptor (RXR) isoforms (α, β, or γ), enhancing its transcriptional activity [28]. Given its pivotal role in gene regulation, dysregulation of VDR signaling has been implicated in a range of pathological conditions, including rickets, psoriasis, renal osteodystrophy, and several autoimmune disorders such as type 1 diabetes, multiple sclerosis, rheumatoid arthritis, and inflammatory bowel disease [29,30].

## 5. Biological Functions of Vit.D

Vit.D exerts a broad range of well-documented biological functions. The active metabolite, 1,25-dihydroxyVit.D [1,25(OH)2D], works in concert with parathyroid hormone (PTH) and calcitonin to regulate calcium and phosphorus homeostasis. This regulation is achieved through enhanced intestinal absorption of these minerals, stimulation of osteoclastic bone resorption, and reduced renal excretion, thereby maintaining skeletal integrity and bone mineralization. Beyond its classical role in calcium–phosphorus metabolism, the widespread expression of VDR in various cell types—including keratinocytes, lymphocytes, pancreatic β-cells, and cells of the pituitary and parathyroid glands—suggests additional biological functions for Vit.D [31]. Vit.D is a key regulator of cellular proliferation and differentiation, influencing multiple cell-specific processes such as cell cycle progression, apoptosis, and differentiation. Notably, 1,25(OH)2D has been implicated in cell cycle arrest at the G0-G1 phase, thereby exerting antiproliferative effects [32]. In one study, the Vit.D analog EB1089 was shown to induce the expression of differentiation-associated epithelial genes, promoting differentiation and reversing the malignant phenotype of squamous cell carcinoma. Additionally, EB1089 inhibited the insulin-like growth factor 1 (IGF-1) signaling pathway, thereby inducing apoptosis in breast cancer cells [32]. Vit.D also functions as a potent immunomodulatory hormone. Several clinical studies have demonstrated that 1,25(OH)2D exerts immunoregulatory effects on both innate and adaptive immune responses, which can be attributed to VDR expression in immune cells and their ability to metabolize Vit.D [33]. Vit.D deficiency has been associated with an increased risk of immune-mediated disorders, including psoriasis, rheumatoid arthritis, type 1 diabetes, sepsis, multiple sclerosis, tuberculosis, and respiratory tract infections [34]. In response to bacterial, viral, or fungal infections, inflammatory cytokines such as interferon-γ and Toll-like receptor activation stimulate macrophages and monocytes to upregulate CYP27B1 expression. This leads to the local conversion of 25(OH)D to its active form, 1,25(OH)2D, which subsequently induces the synthesis of cathelicidin, an endogenous antimicrobial peptide that disrupts microbial membranes and enhances host defense mechanisms [35]. Furthermore, 1,25(OH)2D modulates the function of antigen-presenting cells, such as dendritic cells, by promoting the secretion of immunosuppressive cytokines, thereby contributing to immune tolerance [36]. Several studies have also demonstrated that 1,25(OH)2D directly influences B-cell function in a manner similar to its effects on T cells. Resting B cells exhibit low VDR expression, which increases upon activation. In its active state, 1,25(OH)2D inhibits the differentiation of plasma and memory B cells, induces apoptosis in activated B cells, and promotes the secretion of anti-inflammatory cytokines, thereby modulating humoral immunity [37].

## 6. Vit.D Deficiency and Its Link to Human Diseases

Due to its long half-life and relatively stable serum concentration, independent of PTH fluctuations, the serum level of 25-hydroxyVit.D [25(OH)D] is widely used as a reliable biomarker for assessing whole-body Vit.D status. According to the National Institutes of Health (NIH) and the Office of Dietary Supplements, optimal serum 25(OH)D levels are defined as >20 ng/mL (50 nmol/L), while Vit.D deficiency is diagnosed when serum 25(OH)D levels fall below 12 ng/mL (30 nmol/L) [38]. Vit.D deficiency may arise due to inadequate dietary intake, insufficient sun exposure, malabsorption disorders, or conditions that impair the metabolic activation of Vit.D. Furthermore, several factors influence the risk of Vit.D deficiency, including age, lifestyle, ethnicity, breastfeeding status, geographic latitude, skin pigmentation, and genetic polymorphisms in Vit.D-related genes [39]. Across various regions of the world, Vit.D deficiency remains highly prevalent. Epidemiological studies indicate that more than 20% of the population in India, Pakistan, Afghanistan, Jordan, and Tunisia exhibit serum 25(OH)D levels below 12 ng/mL (30 nmol/L) [40]. A survey revealed that 50% of pregnant women in the United Arab Emirates, 59% of healthy schoolchildren in Saudi Arabia, and 83% of women in Nigeria are Vit.D deficient [41]. A cohort study conducted in Jordan involving 3,007 participants reported that 40.17% were Vit.D deficient, 27.7% had insufficient levels, 17.02% had adequate levels, and only 15.11% had optimal Vit.D levels [42]. Despite high levels of sunlight exposure in both summer and winter, Vit.D deficiency is widespread across Africa and the Middle East. This paradox may be attributed to factors such as sun-avoidant behaviors, traditional clothing that limits skin exposure, dietary restrictions influenced by cultural practices, and genetic variations in Vit.D metabolism [41]. Vit.D deficiency has been strongly associated with various adverse health outcomes, including increased all-cause mortality. A meta-analysis conducted in 2017, encompassing over 17,000 participants, demonstrated a significant correlation between low 25(OH)D levels and elevated mortality risk [43]. Additionally, critically ill patients with sepsis often present with low Vit.D levels. Administration of a single intravenous bolus dose of 400,000 IU of cholecalciferol in the early stages of sepsis has been shown to reduce pro-inflammatory interleukin levels while increasing cathelicidin, an antimicrobial peptide with endotoxin-neutralizing properties [44]. Emerging evidence also suggests that Vit.D deficiency is associated with pregnancy complications such as preeclampsia, low birth weight, and gestational diabetes. Rostami et al. recommend a maintenance dose of 400–600 IU/day of Vit.D supplementation during pregnancy when serum 25(OH)D levels are below 40 ng/mL [45]. Moreover, numerous clinical studies have identified a link between Vit.D deficiency and cardiovascular disorders, including coronary artery disease, cardiomyopathy, and hypertension [46]. It is well established that seasonal influenza outbreaks predominantly occur in winter. One proposed explanation is the seasonal fluctuation in serum 25(OH)D levels due to variations in UV light exposure [47]. Several studies have substantiated this hypothesis, demonstrating that Vit.D deficiency increases susceptibility to acute viral respiratory tract infections in both children and adults [48]. According to data from the National Health and Nutrition Examination Survey (NHANES) in the United States, which included 14,108 participants, individuals with serum 25(OH)D levels below 30 ng/mL had a 58% higher risk of developing acute respiratory infections than those with higher Vit.D levels [48]. Respiratory viruses cause cellular and tissue damage upon entry into the respiratory epithelium, triggering both innate and adaptive immune responses that lead to inflammation and, in severe cases, sepsis, which may be life-threatening [49]. A meta-analysis of 25 randomized controlled trials demonstrated that Vit.D3 or D2 supplementation significantly reduces the risk of acute respiratory infections [50].

## 7. Genetic Variants in Genes Related to Vit.D Metabolism and Signaling

Genetic variants can influence gene regulation, transcription, and protein structure and function, depending on their location within the gene [51]. These variations play a crucial role in explaining inter-individual differences in various phenotypes, including susceptibility to Vit.D deficiency [52]. In this review, we focus on key genes involved in Vit.D metabolism, including Vit.D-activating enzymes CYP2R1 and CYP27B1, the Vit.D-inactivating enzyme CYP24A1, and the VDR.

### 7.1. VDR Genetic Variants

The *VDR* gene, located on chromosome 12q13.11, encodes the Vit.D receptor, a nuclear transcription factor that mediates the biological effects of 1,25(OH)_2_D. Upon activation, VDR heterodimerizes with the retinoid X receptor RXR, forming the VDR/RXR complex, which binds to VDREs in the promoter regions of target genes, thereby regulating their transcription [51].

Single-nucleotide polymorphisms (SNPs) in the *VDR* gene have been implicated in reduced Vit.D activity and are associated with various diseases. Among these, the *rs7975232 (ApaI)*, *rs2228570 (FokI)*, *rs731236 (TaqI)*, and *rs1544410 (BsmI)* polymorphisms are the most extensively studied [51]. *ApaI (rs7975232)* and *BsmI (rs1544410)* are located within intron 8 of the *VDR* gene, while *FokI (rs2228570)* and *TaqI (rs731236)* are situated in exon 2 and exon 9, respectively. The *rs2228570* and *rs731236* polymorphisms may influence translation and alter VDR protein structure, whereas *rs7975232* and *rs1544410* have been associated with mRNA stability and reduced gene expression, ultimately leading to decreased VDR activity and impaired Vit.D function [52].

Numerous studies across different populations have explored the relationship between *VDR* polymorphisms and rheumatoid arthritis (RA). A meta-analysis of 21 studies published before February 2020 found that the *rs2228570* polymorphism exhibited a protective effect against RA in both European and Asian populations, whereas *rs731236* conferred a lower risk of RA among Africans and Arabs. However, *rs1544410* was not significantly associated with RA risk in any population [53]. Another meta-analysis, including 17 studies, examined the association between *VDR* polymorphisms and asthma susceptibility. This study identified a statistically significant link between the wild-type *rs2228570* and homozygous *rs731236* genotypes and asthma susceptibility. Furthermore, the study suggested that ethnic background influences asthma risk, with higher susceptibility observed among American, Asian, and African populations [54].

Several studies have also investigated the role of *VDR* polymorphisms in tuberculosis (TB) susceptibility across diverse ethnic groups. A systematic review of six studies conducted in the Iranian population reported that *rs731236* was significantly associated with increased TB risk across all genetic models, while *rs1544410* was linked to an elevated TB risk only in the dominant genotype model. Conversely, *rs2228570* and *rs7975232* did not show significant associations with TB progression in this population [55]. In addition, a meta-analysis of nine genetic studies published before 2017 examined the association between *VDR* polymorphisms (*rs11568820*, *rs2228570*, *rs731236*, and *rs1544410*) and resistance to enveloped viral infections, including Respiratory Syncytial Virus (RSV), Hepatitis B virus (HBV), and Dengue virus. The findings indicated that *rs2228570* was consistently associated with increased susceptibility to RSV infection, and a global pattern was observed between RSV incidence and the distribution of *rs2228570* alleles, suggesting its potential role as a genetic marker contributing to RSV transmission [54].

In the context of COVID-19, a study conducted in Iran genotyped eight *VDR* polymorphisms (*rs7975232*, *rs1544410*, *rs731236*, *rs2228570*, *rs757343*, *rs739837*, and *rs11568820*) in 500 hospitalized patients using the PCR-RFLP method. The study reported that *rs7975232* was associated with shortness of breath; *rs2228570* with high fever and hypertension; *rs1544410* with chronic kidney disease; and *rs757343* with hypertension, vomiting, and respiratory distress in mild to moderately ill patients [56]. A separate study in Turkey examined the relationship between *VDR* polymorphisms (*rs2228570*, *rs7975232*, *rs731236*, and *rs1544410*) and COVID-19 prognosis using genetic data from 297 COVID-19 patients in the Marmara University Medical Genetics Biobank. The study found that 83% of participants had Vit.D deficiency, with 40.7% exhibiting severe deficiency. Notably, 62.8% of ICU-admitted patients carried the TT genotype of rs731236, highlighting a potential link between this variant and severe COVID-19 outcomes [57]. More recently, Alhammadin et al. (2023) investigated the relationship between VDR gene variants (*rs7975232*, *rs2228570*, and *rs731236*) and COVID-19 severity as well as long-COVID symptoms in 100 Jordanian patients. While rs7975232 and rs2228570 were not significantly associated with disease severity, *rs731236* was significantly linked to milder disease courses, with the wild-type genotype associated with mild illness and the heterozygous genotype predominantly found in asymptomatic individuals. Regarding long-COVID symptoms, the heterozygous *rs7975232* and wild-type *rs731236* genotypes were associated with persistent fatigue and muscle pain, while the homozygous *rs731236* genotype was strongly correlated with prolonged respiratory distress [58].

### 7.2. Genetic Variants in Genes Related to Vit.D Metabolism

The *CYP2R1* gene plays a pivotal role in the Vit.D metabolic pathway. Located on chromosome 11p15.2, this gene spans approximately 15.5 kb and encodes a 501-amino-acid enzyme, 25-hydroxylase, which catalyzes a rate-limiting step in the hepatic conversion of pre-Vit.D into its bioactive form, 25(OH)D [18]. Notably, polymorphisms in *CYP2R1*, such as *rs10741657*, *rs12794714*, and *rs10766197*, have been implicated in modulating 25(OH)D concentrations, likely through alterations in gene expression or enzymatic activity [59]. Several studies have investigated the functional impact of these polymorphisms. A meta-analysis involving 52,417 healthy participants demonstrated that the *rs10741657* variant is significantly associated with lower 25(OH)D levels and an increased risk of Vit.D deficiency under a dominant model (GG + AG vs. AA), particularly among European and Asian populations (OR = 1.42, 95% CI = 1.11–1.83, P = 0.006) [60]. Additionally, a cross-sectional study of 180 patients in Virginia reported that carriers of the *rs10741657* risk allele exhibited a 3.7-fold higher likelihood of Vit.D insufficiency [61]. Another study in individuals with metabolic syndrome identified the G/G homozygous genotype of *rs10741657* as being associated with reduced serum Vit.D3 levels [62].

The *CYP27A1* gene, located on chromosome 2q35, spans approximately 18.6 kb and consists of nine exons and eight introns [63]. Together with CYP2R1 and CYP3A4, *CYP27A1* encodes enzymes involved in the 25-hydroxylation of Vit.D in the liver, a critical step in the biosynthesis of 25(OH)D [7]. Although CYP2R1 exhibits high affinity and specificity for Vit.D, CYP27A1 and CYP3A4 demonstrate broader substrate specificity but a lower affinity for Vit.D metabolism [64]. Among the studied CYP27A1 polymorphisms, *rs17470271* and *rs933994* have been evaluated for their role in Vit.D metabolism [65]. However, their clinical significance remains unclear. A study on pulmonary tuberculosis in a Chinese cohort found no significant association between these variants and Vit.D-related outcomes, suggesting that *CYP27A1* genetic variability alone may not be a primary determinant of Vit.D status [66]. Furthermore, recent research suggests that *CYP27A1* polymorphisms may exert a weaker influence on circulating 25(OH)D levels compared to polymorphisms in *CYP2R1* [67].

The *CYP27B1* gene, located on chromosome 12q14.1, encodes 1-alpha-hydroxylase, a mitochondrial cytochrome P450 enzyme of 508 amino acids, which catalyzes the final activation step of Vit.D by converting 25(OH)D to its biologically active form calcitriol [68]. This hydroxylation occurs primarily in the kidneys and is crucial for Vit.D–mediated calcium homeostasis and immune regulation. The *CYP27B1* gene consists of nine exons and encodes an enzyme integral to renal Vit.D activation [69]. Several key *CYP27B1* polymorphisms, including *rs10877012* and *rs4646536*, have been linked to altered Vit.D levels and associated health outcomes [70]. In a Canadian cohort, rare variants such as *rs118204009 (G/A)*, *rs118204011 (C/T)*, and *rs118204012 (A/G)* were found to correlate with lower Vit.D levels and conditions such as VDDT1 rickets. Specifically, individuals homozygous for the *rs118204012 (AA)* genotype exhibited a significantly increased risk of Vit.D insufficiency, particularly among males [71].

Further research has explored the association between *CYP27B1* polymorphisms and autoimmune diseases. The *rs10877012* polymorphism has been reported to influence serum calcidiol levels, potentially modulating immune responses in disorders such as multiple sclerosis, RA, and systemic lupus erythematosus. This suggests that genetic variations in *CYP27B1* may impact Vit.D metabolism and immune function, indicating the gene’s role in disease susceptibility and Vit.D homeostasis [72]. Additionally, a study on Iranian populations revealed a significant association between the *rs4646536* variant and Vit.D deficiency, with carriers displaying a markedly higher risk of Vit.D insufficiency [70].

### 7.3. Genetic Variants in Vit.D-Inactivation Gene

The *CYP24A1* gene, located on chromosome 20q13.2, encodes 24-hydroxylase, a mitochondrial enzyme essential for the inactivation of Vit.D [73]. This enzyme catalyzes the 24-hydroxylationof 1,25(OH)_2_D, converting it into calcitroic acid, an inactive metabolite that is subsequently excreted, thereby tightly regulating Vit.D homeostasis [74]. *CYP24A1* is highly expressed in the kidney and plays a crucial role in increasing the solubility of Vit.D metabolites for renal clearance [75,76]. Several SNPs in *CYP24A1*, including *rs2248137*, *rs2296241*, and *rs927650*, have been associated with circulating Vit.D levels and disease susceptibility [77,78]. One study reported a significant association between the *rs2248137* variant and multiple sclerosis risk, showing that MS patients carrying the CC genotype had significantly lower 25(OH)D levels compared to individuals with the GG or CG genotypes [64]. In addition, *CYP24A1* polymorphisms have been implicated in non-alcoholic fatty liver disease (NAFLD) and Vit.D deficiency. A study identified a strong correlation between the *rs2296241* and *rs2248359* variants and reduced serum Vit.D levels, suggesting that genetic variations in *CYP24A1* may contribute to impaired Vit.D metabolism in patients with NAFLD [79]. Further research has explored the role of *CYP24A1* variants in differentiated thyroid cancer (DTC). A study in a German cohort found that specific *CYP24A1* haplotypes, such as *rs2248137C/rs2296241G*, were significantly associated with lower circulating 1,25(OH)_2_D₃ levels in DTC patients compared to healthy controls [80]. These findings suggest that genetic variants in *CYP24A1* may contribute to alterations in Vit.D metabolism, hence influencing the risk and progression of certain diseases.

## 8. In Silico Analysis of Genetic Variants Related to Vit.D Metabolism and Signaling

We conducted an in silico evaluation, which refers to computational simulations and bioinformatics tools used to analyze biological data of nonsynonymous genetic variants in key human Vit.D-related genes—*CYP2R1*, *CYP27B1*, *CYP24A1*, and *VDR*—identified in the clinical variant database of GenBank (https://www.ncbi.nlm.nih.gov/clinvar/ accessed on August 2024). The analyzed genetic variants, using in silico tools, are genetic variants with unknown or uncertain functionality on human Vit.D deficiency. The analysis utilized widely adopted computational prediction tools, PolyPhen-2 and Sorting Intolerant From Tolerant (SIFT), to assess the potential functional impact of these variants. PolyPhen-2 predicts the potential impact of amino acid substitutions by analyzing sequence conservation and structural changes within the protein. It assigns scores based on the likelihood of a variant affecting protein function [81]. Similarly, SIFT evaluates evolutionary conservation, determining whether an amino acid substitution is likely to be tolerated or damaging based on sequence homology and amino acid properties [82].

The results for *CYP2R1*, *CYP27B1*, *CYP24A1*, and *VDR* variants are summarized in Table 1, Table 2, Table 3 and Table 4, respectively. Notably, the functional consequences of many of these variants remain uncharacterized in vitro and clinically. The in silico assessment of *CYP2R1* nonsynonymous variants (Table 1) revealed a broad spectrum of predicted functional effects. Variants such as *Pro36Leu (107C>T)*, *Pro41Thr (121C>A)*, *Ile332Thr (995T>C)*, *Leu300Arg (899T>G)*, *Gly450Arg (1348G>C)*, and *Arg248Ser (744A>C)* were predicted to be deleterious by both tools. PolyPhen-2 assigned scores approaching or equal to 1.0, suggesting a high likelihood of pathogenicity, while SIFT classified them as “not tolerated.” These substitutions likely disrupt protein structure or function, as they occur at residues critical for enzymatic activity or stability. Conversely, several variants were consistently predicted to be benign. For instance, *Glu8Lys (22G>A)*, *Arg67Lys (200G>A)*, and *Thr402Ile (1205C>T)* were classified as having minimal or no impact on protein function by both tools. However, discrepancies between prediction tools were observed for certain variants. For example, *Leu193Met* was classified as “damaging” by PolyPhen-2(score 0.963) but deemed “tolerated” by SIFT. These inconsistencies likely arise from differences in the computational algorithms and training parameters used by each tool [83]. When conflicting results occur, researchers can consider additional factors such as population-specific allele frequencies and functional assays.

Although in silico tools offer useful predictions about the possible effects of genetic variants, care should be taken when interpreting their findings. Discrepancies between these tools indicate the need for experimental validation to confirm functional effects.

Similarly, the analysis of *CYP27B1* variants (Table 2) showed that among the 100 examined variants, *Arg14Cys (40C>T)*, *Arg389Cys (1165C>T)*, *Arg459Leu (1376G>T)*, and *Arg335Pro (1004G>C)* were consistently classified as deleterious by both PolyPhen-2 (high pathogenicity scores) and SIFT (“not tolerated”). These variants are predominantly located at conserved residues, frequently involving polar or charged amino acids essential for CYP27B1 stability and enzymatic function. In contrast, substitutions such *as Pro112Leu (335C>T)*, *Ala129Thr (385G>A)*, and *Gly208Val (623G>T)* were predicted to be benign, suggesting negligible effects on protein function.

The evaluation of *CYP24A1* variants (Table 3) indicated diverse functional consequences. For example, *Met1Ile (3G>T)* was predicted to be damaging by PolyPhen-2 (score 0.931) and “not tolerated” by SIFT (score 0.30), indicating a likely deleterious effect. Similarly, *Gly102Arg (304G>C)* was classified as highly damaging by both tools, with a PolyPhen-2score of 1.0 and a SIFT score of 1, suggesting a severe impact on protein function. Conversely, *Ser8Gly (22A>G)* was predicted to be benign, with a PolyPhen-2 score of 0.104 and a SIFT score of 0.70, implying minimal disruption. Variants such as *Asp84Asn (250G>A)* and *Val218Leu (652G>T)* were similarly classified as benign. However, conflicting predictions were observed for *Ala12Pro (34G>C)*, which was considered “damaging” by PolyPhen-2 (score 0.612) but “tolerated” by SIFT (score 0.70).

Finally, the analysis of *VDR* variants (Table 4) identified several substitutions classified as deleterious, with PolyPhen-2 scores nearing 1.0 and “not tolerated” designations by SIFT, indicating a high probability of functional impairment. Notable examples include *Arg22Trp (64C>T)*, *Arg54Trp (160C>T)*, and *Val346Met (1036G>A)*. In contrast, *Met4Val (10A>G)* and *Ala6Val (17C>T)* were consistently classified as benign, with low PolyPhen-2 scores and “tolerated” designations in SIFT, suggesting minimal functional consequences.

## 9. Genetic Variants Identified in Human Clinical Studies

Table 5 summarizes genetic variants in Vit.D-metabolizing genes that have been associated with various diseases in human studies. Notably, the majority of disease-associated mutations in Vit.D -related genes are nonsense SNPs, which introduce premature stop codons, leading to truncated, non-functional proteins that impair Vit.D metabolism and function. Among these variants, the *CYP2R1* promoter variant rs10741657 has been linked to reduced circulating 25-hydroxyVit.D levels, increasing susceptibility to Vit.D deficiency, particularly in White European populations. In the *CYP27B1* gene, missense variants have been implicated in various forms of VDDR. The *rs118204009* variant, which results in an arginine-to-histidine (Arg389His) substitution, has been clinically associated with VDDR1A, causing impaired calcium homeostasis and bone mineralization, as supported by both functional studies and in silico predictions (Table 2). Similarly, the *rs118204011* variant, causing a leucine-to-phenylalanine (Leu343Phe) substitution, is linked to VDDR1 and altered enzymatic function. Meanwhile, the *rs118204012* variant, which replaces glutamic acid with glycine (Glu189Gly), has been associated with VDDR1A, although in silico analysis suggests it may have a benign impact on protein function (Table 2).

## 10. Conclusions

Vit.D deficiency and its associated disorders are widespread, affecting populations across various regions globally. Both genetic and non-genetic factors contribute to individual variability in Vit.D metabolism, signaling, and response. Our in silico analysis of genetic variants in Vit.D-related genes with unknown functionality demonstrates that numerous variants significantly impact the key proteins involved in Vit.D activation, inactivation, and signaling mechanisms. These findings may indicate the polygenic nature of Vit.D response, suggesting that analyzing a single genetic variant may not fully explain the phenotypic variability observed in Vit.D deficiency. In this review, we have systematically compiled and examined genetic variants associated with Vit.D synthesis, metabolism, and signaling, integrating in silico functional predictions with clinically studied human variants. This comprehensive approach provides a more detailed understanding of the molecular basis of Vit.D function. A comprehensive analysis of all Vit.D-related genes is essential for advancing personalized medicine in the context of Vit.D deficiency. The development of a specialized diagnostic panel incorporating key genetic variants with significant functional impact on Vit.D metabolism and activity would be invaluable for precise risk assessment and the implementation of personalized treatment strategies. Such advancements could pave the way for targeted interventions to mitigate the adverse health effects associated with Vit.D deficiency.

## 11. Strengths and Limitations

This review highlights its strong points. It offers a thorough review of the literature on genetic polymorphisms linked to Vit.D and their possible effects on biochemical and metabolic parameters. Finding these genetic variations can aid in the development of individualized treatment plans for patients with Vit.D deficiency, making the review clinically relevant.

It is important to recognize the limitations of this review. Because studies that report significant associations are more likely to be published, potential publication bias may be an issue. Furthermore, there is a lack of thorough information on some ethnic groups, such as Middle Eastern populations. Lastly, a lot of research does not completely take into consideration confounding variables like diet, exercise, and sun exposure, all of which can influence Vit.D levels.

## Figures and Tables

**Table 1:** Genetic variants in *CYP2R1* gene and their functionality prediction using in silico tools.

Genetic Variant ID	Location	Amino Acid Substitution	PolyPhen		SIFT	
1592939	4T>C	Trp2Arg	Benign	0	Not tolerated	0.07
2179637	14G>C	Trp5Ser	Benign	0	Not tolerated	0.21
1919558	17G>C	Arg6Thr	Benign	0	Tolerated	0.36
1399245	22G>A	Glu8Lys	Benign	0.018	Tolerated	0.36
792888	29G>C	Gly10Ala	Benign	0.001	Tolerated	0.50
2084757	40C>G	Leu14Val	Benign	0.295	Tolerated	0.30
1414730	69C>A	Phe23Leu	Benign	0	Tolerated	0.07
3270606	77G>C	Gly26Ala	Benign	0	Tolerated	0.29
1059990	107C>T	Pro36Leu	Damaging	0.945	Not tolerated	1
1939745	112G>A	Gly38Ser	Benign	0.07	Tolerated	0
2414155	121C>A	Pro41Thr	Damaging	1	Not tolerated	1
2332099	163C>G	Leu55Val	Damaging	0.86	Tolerated	0.07
1950230	169G>T	Ala57Ser	Benign	0	Tolerated	0.14
1368104	200G>A	Arg67Lys	Benign	0	Tolerated	0
2533332	203A>C	Lys68Thr	Benign	0.042	Tolerated	0
847396	235T>A	Leu79Ile	Benign	0.036	Not tolerated	1
1345679	253T>C	Ser85Pro	Benign	0	Tolerated	0
1912325	272G>A	Gly91Asp	Damaging	0.94	Not tolerated	1
2134	296T>C	Leu99Pro	Damaging	1	Not tolerated	1
960805	310G>A	Glu104Lys	Damaging	0.984	Not tolerated	1
2068341	325A>G	Arg109Gly	Damaging	1	Not tolerated	1
1000400	352A>G	Met118Val	Benign	0.43	Tolerated	0
957411	421G>A	Val141Ile	Benign	0.003	Tolerated	0
934633	467C>G	Ser156Cys	Damaging	0.454	Not tolerated	1
2374820	497A>G	Asn166Ser	Benign	0.001	Tolerated	0
835914	515A>G	Tyr172Cys	Benign	0.07	Tolerated	0
1304683	551C>T	Thr184Met	Damaging	0.777	Not tolerated	1
1310664	577C>A	Leu193Met	Damaging	0.963	Tolerated	0
2060962	577C>G	Leu193Val	Benign	0.002	Tolerated	0
2172123	582C>G	Ile194Met	Damaging	0.558	Not tolerated	1
2067369	661G>A	Ala221Thr	Benign	0.294	Tolerated	0
1918882	744A>C	Arg248Ser	Benign	0.17	Tolerated	0
1996774	806A>G	Lys269Arg	Benign	0.034	Tolerated	0
2281495	850A>G	Met284Val	Benign	0.077	Tolerated	0
418154	851T>C	Met284Thr	Benign	0.059	Not tolerated	1
218799	852G>A	Met284Ile	Benign	0.00	Tolerated	0
1435363	859G>A	Gly287Ser	Benign	0.00	Tolerated	0
2470790	899T>G	Leu300Arg	Damaging	1	Not tolerated	1
1356309	913G>A	Gly305Ser	Benign	0.166	Tolerated	0
936742	950A>G	Asn317Ser	Damaging	0.864	Tolerated	0
429315	995T>C	Ile332Thr	Damaging	0.999	Not tolerated	1
1059302	1011G>T	Gln337His	Benign	0.002	Tolerated	0
1714746	1054T>A	Trp352Arg	Damaging	0.876	Not tolerated	1
429316	1126C>T	Pro376Ser	Damaging	1	Not tolerated	1
2160764	1142A>G	His381Arg	Damaging	0.725	Tolerated	0
2067311	1147A>G	Thr383Ala	Damaging	0.909	Tolerated	0
3079576	1148C>T	Thr383Ile	Damaging	0.998	Tolerated	0
3079577	1151C>G	Ser384Cys	Damaging	0.999	Not tolerated	1
1008903	1166T>A	Val389Glu	Damaging	0.999	Not tolerated	1
2288720	1178C>T	Ser393Phe	Benign	0.11	Tolerated	0
930898	1181T>C	Ile394Thr	Damaging	1	Not tolerated	1
2260893	1196C>G	Thr399Arg	Damaging	0.511	Tolerated	0
1302522	1198G>C	Val400Leu	Damaging	0.662	Tolerated	0
2416170	1205C>T	Thr402Ile	Benign	0.20	Tolerated	0
2092937	1232A>G	Glu411Gly	Damaging	0.992	Tolerated	0
2193872	1280A>G	Asp427Gly	Damaging	0.984	Not tolerated	1
2263146	1291T>C	Tyr431His	Benign	0.001	Tolerated	0
2964676	1298C>G	Ala433Gly	Benign	0.005	Tolerated	0
2554404	1303A>G	Lys435Glu	Damaging	0.593	Tolerated	0
3016792	1322T>C	Phe441Ser	Damaging	1	Not tolerated	1
1025990	1348G>C	Gly450Arg	Damaging	1	Not tolerated	1
2138093	1351G>A	Glu451Lys	Damaging	0.994	Not tolerated	1
1385510	1363C>T	Arg455Trp	Damaging	1	Not tolerated	1
1391635	1364G>A	Arg455Gln	Damaging	1	Not tolerated	1
1334316	1394T>G	Leu465Trp	Damaging	1	Not tolerated	1
2233339	1397T>C	Leu466Pro	Damaging	1	Not tolerated	1
3079578	1424A>G	His475Arg	Benign	0	Tolerated	0.50
2580540	1427A>T	Glu476Val	Benign	0	Not tolerated	0.50
2127312	1436C>A	Pro479Gln	Damaging	1	Tolerated	0
3270607	1478C>G	Pro493Arg	Damaging	0.986	Tolerated	0

PolyPhen provides a probability score for how damaging a variant is, with higher values indicating more likelihood of pathogenicity, while SIFT predicts whether a substitution is “tolerated” or “not tolerated”, with a focus on protein functionality. The numbering of nucleotides is based on the DNA sequence of the *CYP2R1* transcript NM_024514.5.

**Table 2:** Genetic variants in *CYP27B1* gene and their functionality prediction using in silico tools.

Genetic Variant ID	Location	Amino Acid Substitution	PolyPhen		SIFT	
310004	40C>T	.Arg14Cys	Damaging	0.996	Not tolerated	0.73
934616	41G>A	Arg14His	Damaging	0.989	Not tolerated	0.73
3079447	49T>A	Trp17Arg	Benign	0	Tolerated	0.23
1943189	148G>A	Ala50Thr	Benign	0	Tolerated	0.09
1715471	163A>G	Lys55Glu	Damaging	1	Not tolerated	0.91
1467327	164A>T	Lys55Met	Damaging	0.996	Not tolerated	0.91
1457495	170G>T	Gly57Val	Damaging	0.996	Tolerated	0.09
2301172	200A>G	Gln67Arg	Benign	0.016	Tolerated	0.09
1975900	230T>C	Leu77Pro	Benign	0.016	Tolerated	0.09
3079444	286G>A	Glu96Lys	Benign	0.085	Tolerated	0.09
380287	305G>A	Gly102Glu	Benign	0.014	Tolerated	0.09
2180339	310C>T	Arg104Trp	Benign	0.004	Tolerated	0.09
1960647	311G>A	Arg104Gln	Benign	0.175	Tolerated	0.09
1658	320G>A	Arg107His	Damaging	1	Not tolerated	0.91
1923855	328T>C	Phe110Leu	Benign	0	Tolerated	0.09
1705730	335C>T	Pro112Leu	Benign	0	Tolerated	0.09
2191011	346C>G	His116Asp	Damaging	1	Not tolerated	0.91
2092360	350G>A	Arg117His	Damaging	1	Not tolerated	0.91
1950321	358C>T	Arg120Cys	Damaging	1	Not tolerated	0.91
432037	373G>A	Gly125Arg	Damaging	1	Not tolerated	0.91
1659	374G>A	Gly125Glu	Damaging	1	Not tolerated	0.91
722611	385G>A	Ala129Thr	Damaging	0.576	Tolerated	0.09
1339453	386C>T	Ala129Val	Damaging	0.998	Tolerated	0.09
1016722	413G>T	Arg138Leu	Damaging	1	Not tolerated	1
1067732	428C>T	Pro143Leu	Damaging	0.585	Tolerated	0
310002	437T>A	Leu146His	Damaging	1	Not tolerated	1
2514871	448G>A	Ala150Thr	Benign	0.295	Tolerated	0
3079445	461A>T	Tyr154Phe	Benign	0.190	Tolerated	0
3270536	463G>T	Ala155Ser	Benign	0	Tolerated	0
3339156	490G>A	Asp164Asn	Damaging	1	Not tolerated	1
1443676	511C>T	Arg171Cys	Damaging	1	Not tolerated	1
310001	541G>T	Ala181Ser	Benign	0	Tolerated	0.27
1345874	547G>C	Val183Leu	Damaging	1	Not tolerated	1
1674	566A>G	Glu189Gly	Benign	0.165	Tolerated	0
3079449	571T>C	Tyr191His	Benign	0.253	Tolerated	0
2152155	580G>A	Gly194Arg	Benign	0	Tolerated	0
968805	584T>A	Leu195Gln	Damaging	1	Not tolerated	1
1339454	623G>T	Gly208Val	Damaging	1	Not tolerated	1
2336628	651C>A	Asp217Glu	Benign	0	Tolerated	0
2187188	657G>C	Glu219Asp	Benign	0.176	Tolerated	0
1708812	704C>A	Thr235Asn	Damaging	1	Not tolerated	1
2332756	707T>C	Met236Thr	Benign	0.025	Tolerated	0
2116143	733C>T	Leu245Phe	Benign	0.01	Tolerated	0
1900220	764G>A	Arg255Gln	Benign	0	Tolerated	0
2627673	779T>G	Met260Arg	Damaging	1	Not tolerated	1
2499531	781T>G	Phe261Val	Damaging	1	Not tolerated	1
310000	788T>G	Phe263Cys	Damaging	1	Not tolerated	1
309999	794A>T	Gln265Leu	Damaging	1	Not tolerated	1
2123967	850G>A	Glu284Lys	Benign	0.01	Tolerated	0.09
2911178	914A>C	Gln305Pro	Benign	0	Tolerated	0
1929411	939G>C	Glu313Asp	Damaging	1	Not tolerated	1
1666	962C>G	Thr321Arg	Damaging	1	Not tolerated	1
1660	1004G>C	Arg335Pro	Damaging	1	Not tolerated	1
3270537	1009C>T	Pro337Ser	Damaging	1	Not tolerated	1
1673	1027C>T	Leu343Phe	Damaging	1	Not tolerated	1
2359237	1052T>G	Leu351Arg	Damaging	1	Not tolerated	1
3362736	1052T>C	Leu351Pro	Damaging	1	Not tolerated	1
2191446	1094C>T	Ser365Phe	Benign	0	Tolerated	0
2020701	1108C>A	Leu370Met	Damaging	1	Not tolerated	1
1339455	1160A>C	Asn387Thr	Damaging	0.774	Tolerated	0
1672	1165C>G	Arg389Gly	Damaging	1	Not tolerated	1
1324206	1165C>T	Arg389Cys	Damaging	1	Not tolerated	1
1669	1166G>A	.Arg389His	Damaging	1	Not tolerated	1
2806255	1192G>A	Gly398Ser	Benign	0.41	Tolerated	0
1001177	1198T>G	Tyr400Asp	Damaging	1	Not tolerated	1
1936106	1217C>T	Thr406Met	Damaging	1	Not tolerated	1
1343091	1232G>A	Cys411Tyr	Damaging	0.965	Not tolerated	1
265095	1286G>C	Arg429Pro	Damaging	0.976	Tolerated	0
2735903	1294C>T	Arg432Cys	Damaging	1	Not tolerated	1
2331380	1318C>T	Pro440Ser	Benign	0.025	Tolerated	0
3079443	1337T>G	Leu446Arg	Damaging	1	Not tolerated	1
2722314	1352G>T	Gly451Val	Damaging	1	Not tolerated	1
802871	1357C>T	Arg453Cys	Damaging	1	Not tolerated	1
2103440	1364G>A	Cys455Tyr	Damaging	1	Not tolerated	1
1007304	1376G>A	Arg459His	Damaging	1	Not tolerated	1
3251601	1376G>T	Arg459Leu	Damaging	1	Not tolerated	1
2808964	1382C>A	Ala461Glu	Damaging	1	Not tolerated	1
309996	1385A>T	Glu462Val	Damaging	1	Not tolerated	1
857514	1474C>T	Arg492Trp	Damaging	0.996	Tolerated	0
2164587	1499G>A	Ser500Asn	Damaging	0.599	Tolerated	0
309995	1505A>G	Asn502Ser	Benign	0.238	Tolerated	0
2099009	1517T>G	Leu506Trp	Damaging	0.923	Tolerated	0.09

PolyPhen provides a probability score for how damaging a variant is, with higher values indicating more likelihood of pathogenicity, while SIFT predicts whether a substitution is “tolerated” or “not tolerated”, with a focus on protein functionality. The numbering of nucleotides is based on the DNA sequence of the *CYP27B1* transcript NM_000785.4.

**Table 3:** Genetic variants in *CYP24A1* gene and their functionality prediction using in silico tools.

Genetic Variant ID	Location	Amino Acid Substitution	PolyPhen		SIFT	
694500	3G>T	Met1Ile	Damaging	0.931	Not tolerated	0.30
1446612	22A>G	Ser8Gly	Benign	0.104	Tolerated	0.70
3079403	34G>C	Ala12Pro	Damaging	0.612	Tolerated	0.70
1530538	37G>A	Ala13Thr	Benign	0.008	Not tolerated	0.30
338839	73C>G	Pro25Ala	Benign	0.049	Not tolerated	0.30
3270510	78A>C	Arg26Ser	Benign	0.024	Not tolerated	0.30
338838	101C>T	Thr34Met	Damaging	0.661	Tolerated	0.70
2154023	116G>T	Arg39Leu	Benign	0.208	Not tolerated	0.30
3079401	116G>A	Arg39Gln	Benign	0.226	Tolerated	0.70
2311515	134C>A	Pro45Gln	Benign	0.078	Not tolerated	0.30
1021961	175C>T	Pro59Ser	Damaging	0.98	Not tolerated	0.70
338836	217A>T	Ile73Phe	Damaging	0.967	Tolerated	0.1
1921257	250G>A	Asp84Asn	Benign	0.002	Tolerated	0.1
338833	295A>G	Met99Val	Damaging	0.998	Tolerated	0
897021	296T>C	Met99Thr	Damaging	0.996	Tolerated	0
2124827	304G>C	Gly102Arg	Damaging	1	Not tolerated	1
2652423	305G>C	Gly102Ala	Damaging	1	Not tolerated	1
1384043	313G>C	Glu105Gln	Benign	0.428	Tolerated	0
1974478	320T>C	Val107Ala	Damaging	1	Not tolerated	1
2398535	323A>G	His108Arg	Damaging	0.997	Tolerated	0
1988031	324C>A	His108Gln	Damaging	0.991	Tolerated	0
2490932	337T>G	Cys113Gly	Benign	0.003	Tolerated	0
1044874	343C>A	Leu115Met	Damaging	0.988	Tolerated	0
2367135	356A>G	Tyr119Cys	Damaging	1	Not tolerated	1
338832	359G>T	Arg120Leu	Damaging	1	Not tolerated	1
3019023	359G>A	Arg120His	Damaging	1	Not tolerated	1
2101959	366G>T	Glu122Asp	Damaging	1	Not tolerated	1
2477504	368G>A	Ser123Asn	Benign	0.339	Tolerated	0
897020	376C>T	Pro126Ser	Damaging	1	Not tolerated	1
2866341	382C>T	Arg128Trp	Damaging	1	Not tolerated	1
728557	385C>A	Leu129Met	Damaging	0.999	Tolerated	0
897019	397C>G	Pro133Ala	Damaging	1	Tolerated	0
1067888	400T>G	Trp134Gly	Damaging	1	Not tolerated	1
1036471	425A>G	Lys142Arg	Benign	0.039	Tolerated	0
2729394	437G>A	Gly146Glu	Damaging	1	Not tolerated	1
631878	443T>C	Leu148Pro	Damaging	0.998	Not tolerated	1
1489324	457G>A	Glu153Lys	Benign	0	Tolerated	0
1038891	467A>C	Gln156Pro	Damaging	0.96	Tolerated	0
285894	469C>T	Arg157Trp	Damaging	0.999	Not tolerated	1
1373583	469C>G	Arg157Gly	Damaging	0.997	Not tolerated	1
634948	470G>A	Arg157Gln	Damaging	0.929	Tolerated	0
895613	473T>C	Val158Ala	Benign	0.199	Not tolerated	1
942179	475C>T	Arg159Trp	Damaging	1	Not tolerated	1
29676	476G>A	Arg159Gln	Damaging	1	Not tolerated	1
935088	505C>A	Pro169Thr	Damaging	0.565	Not tolerated	1
1443102	533A>G	Lys178Arg	Benign	0.008	Tolerated	0
3079404	571G>T	Asp191Tyr	Damaging	1	Not tolerated	1
2185427	576G>C	Glu192Asp	Benign	0.001	Tolerated	0
338830	577C>A	Leu193Ile	Benign	0.036	Tolerated	0
2495690	581G>A	Cys194Tyr	Damaging	0.956	Tolerated	0
1358313	598G>A	Val200Ile	Benign	0	Tolerated	0
338829	604G>C	Asp202His	Damaging	0.981	Not tolerated	1
1079881	616G>A	Glu206Lys	Damaging	0.999	Tolerated	0
2652422	625A>G	Lys209Glu	Damaging	1	Tolerated	0
1948727	639A>C	Glu213Asp	Damaging	0.997	Not tolerated	1
2067418	652G>T	Val218Leu	Benign	0.131	Tolerated	0
2683548	652G>A	Val218Met	Damaging	0.993	Not tolerated	1
1948222	683A>G	Gln228Arg	Benign	0.002	Not tolerated	1
2411429	688A>C	Asn230His	Damaging	0.858	Tolerated	0
2544280	688A>T	Asn230Tyr	Damaging	0.948	Not tolerated	1
338827	695G>A	Gly232Glu	Benign	0.001	Tolerated	0
898600	735G>A	Met245Ile	Damaging	0.785	Tolerated	0
1979274	743C>T	Thr248Met	Damaging	0.731	Not tolerated	1
338824	776T>C	Leu259Pro	Damaging	1	Tolerated	0.10
1913130	788T>C	Leu263Pro	Damaging	1	Not tolerated	0.90
1464834	815C>G	Thr272Ser	Damaging	1	Tolerated	0
1028372	833T>C	Ile278Thr	Damaging	1	Not tolerated	1
2549031	856A>G	Ile286Val	Benign	0.003	Tolerated	0
1406180	859G>A	Asp287Asn	Benign	0.013	Tolerated	0
338823	861C>A	Asp287Glu	Benign	0.085	Tolerated	0
1015029	904C>T	Leu302Phe	Damaging	0.996	Tolerated	0
697520	908G>C	Cys303Ser	Damaging	0.614	Tolerated	0
1945084	928C>T	Arg310Trp	Damaging	0.989	Tolerated	0
2664932	948G>T	Leu316Phe	Damaging	0.998	Tolerated	0
29681	964G>A	Glu322Lys	Damaging	1	Not tolerated	1
1018075	989C>T	Thr330Met	Damaging	1	Not tolerated	1
1647156	1031G>A	Arg344His	Damaging	1	Not tolerated	1
1959393	1058T>C	Leu353Pro	Damaging	1	Tolerated	0
2081156	1100G>A	Arg367Gln	Benign	0.184	Tolerated	0
1464180	1103C>A	Ala368Glu	Damaging	0.997	Tolerated	0
338817	1121T>C	Met374Thr	Damaging	0.874	Not tolerated	1
338816	1124C>T	Pro375Leu	Damaging	1	Not tolerated	1
2040775	1139G>A	Cys380Tyr	Damaging	1	Not tolerated	1
1455164	1147G>C	Glu383Gln	Damaging	1	Not tolerated	1
29679	1186C>T	Arg396Trp	Damaging	1	Not tolerated	1
953906	1187G>A	Arg396Gln	Damaging	1	Not tolerated	1
338814	1207G>A	Val403Ile	Damaging	0.618	Tolerated	0
896943	1219T>A	Tyr407Asn	Damaging	1	Not tolerated	1
29680	1226T>C	Leu409Ser	Damaging	1	Not tolerated	1
2077753	1235G>A	Gly412Glu	Damaging	1	Tolerated	0
3362760	1238C>A	Thr413Lys	Damaging	0.612	Not tolerated	1
2005956	1259A>C	Gln420Pro	Damaging	0.996	Tolerated	0
1333737	1268G>T	Gly423Val	Damaging	1	Tolerated	0
1904576	1282A>G	Asn428Asp	Damaging	0.812	Tolerated	0
2558744	1288G>A	Glu430Lys	Benign	0.001	Tolerated	0
2535527	1298G>T	Ser433Ile	Damaging	0.601	Not tolerated	1
1333645	1310C>A	Pro437His	Damaging	1	Not tolerated	1
931571	1315C>T	Arg439Cys	Damaging	1	Not tolerated	1
338813	1361C>T	Pro454Leu	Damaging	1	Not tolerated	1
1354397	1366G>C	Gly456Arg	Damaging	1	Not tolerated	1
896940	1369G>A	Val457Ile	Benign	0.003	Tolerated	0
1984362	1387A>G	Ile463Val	Benign	0.023	Tolerated	0
2716855	1390G>C	Gly464Arg	Damaging	1	Not tolerated	1
1063076	1394G>A	Arg465His	Damaging	1	Not tolerated	1
2454835	1450G>A	Asp484Asn	Benign	0.002	Tolerated	0
1622534	1460C>T	Ala487Val	Damaging	1	Tolerated	0
2482164	1467C>G	Asp489Glu	Damaging	0.999	Tolerated	0
1919479	1490A>C	His497Pro	Damaging	1	Tolerated	0
3079402	1502T>A	Leu501Gln	Damaging	1	Not tolerated	1
3066341	1507C>G	Pro503Ala	Damaging	1	Not tolerated	1
2138356	1508C>T	Pro503Leu	Damaging	1	Not tolerated	1
895540	1513C>G	Arg505Gly	Damaging	1	Not tolerated	1
3016767	1513C>T	Arg505Trp	Damaging	1	Not tolerated	1
895539	1518A>T	Glu506Asp	Damaging	0.944	Tolerated	0
1446233	1518A>C	Glu506Asp	Damaging	0.944	Tolerated	0
1903225	1519C>A	Leu507Ile	Damaging	1	Tolerated	0
1803423	1525A>G	Ile509Val	Benign	0.002	Tolerated	0
895538	1528G>A	Ala510Thr	Damaging	0.921	Tolerated	0
697436	1529C>T	Ala510Val	Benign	0.265	Tolerated	0
2327122	1535G>A	Cys512Tyr	Benign	0.015	Tolerated	0.1

PolyPhen provides a probability score for how damaging a variant is, with higher values indicating more likelihood of pathogenicity, while SIFT predicts whether a substitution is “tolerated” or “not tolerated”, with a focus on protein functionality. The numbering of nucleotides is based on the DNA sequence of the CYP24A1 transcript NM_000782.5.

**Table 4:** Genetic variants in *VDR* gene and their functionality prediction using in silico tools.

Genetic Variant ID	Location	Amino Acid Substitution	Polyphen		SIFT	
308887	2T>C	Met1Thr	Benign	0.29	Not tolerated	0.68
3235711	2T>G	Met1Arg	Damaging	0.97	Not tolerated	0.68
2174849	10A>G	Met4Val	Benign	0	Tolerated	0.42
882357	14C>T	Ala5Val	Benign	0	Tolerated	0.47
2261491	17C>T	Ala6Val	Benign	0	Tolerated	0.47
882356	52C>T	Arg18Trp	Damaging	0.99	Not tolerated	0.53
3032901	53G>A	Arg18Gln	Benign	0.01	Tolerated	0.47
2410921	58G>A	Val20Met	Benign	0.07	Tolerated	0.47
959736	61C>T	Pro21Ser	Damaging	0.96	Not tolerated	0.74
882354	64C>T	Arg22Trp	Damaging	1	Not tolerated	1
880999	65G>A	Arg22Gln	Damaging	0.91	Not tolerated	1
2072618	65G>C	Arg22Pro	Damaging	1	Not tolerated	1
2137321	76G>A	Val26Met	Damaging	1	Not tolerated	1
2759116	79T>C	Cys27Arg	Damaging	1	Not tolerated	1
950224	86A>G	Asp29Gly	Damaging	1	Not tolerated	1
848467	89G>A	Arg30Gln	Damaging	0.61	Tolerated	0
7745	98G>A	Gly33Asp	Damaging	1	Not tolerated	1
3339539	98G>C	Gly33Ala	Damaging	1	Not tolerated	1
1407165	110A>G	Asn37Ser	Damaging	0.99	Not tolerated	1
7753	137G>A	Gly46Asp	Damaging	1	Not tolerated	1
1954983	137G>C	Gly46Ala	Damaging	0.99	Not tolerated	1
7750	149G>A	Arg50Gln	Damaging	1	Not tolerated	1
1193364	156G>T	Met52Ile	Benign	0.11	Tolerated	0
3004611	160C>T	Arg54Trp	Damaging	1	Not tolerated	1
1370955	161G>A	Arg54Gln	Damaging	0.99	Not tolerated	1
717364	176C>T	Thr59Ile	Benign	0.22	Tolerated	0
961739	182C>T	Pro61Leu	Damaging	0.94	Not tolerated	1
1957857	191G>A	Gly64Glu	Damaging	0.54	Not tolerated	1
1425408	199C>T	Arg67Cys	Damaging	0.81	Not tolerated	1
1009301	200G>A	Arg67His	Benign	0.001	Tolerated	0
1059781	212A>G	Asp71Gly	Damaging	0.58	Tolerated	0
7746	218G>A	Arg73Gln	Damaging	1	Not tolerated	1
3148965	220C>A	Arg74Ser	Damaging	0.999	Not tolerated	1
880998	221G>A	Arg74His	Damaging	1	Not tolerated	1
635013	227G>T	Cys76Phe	Damaging	1	Not tolerated	1
3188427	236G>A	Cys79Tyr	Damaging	1	Not tolerated	1
7749	239G>A	Arg80Gln	Damaging	0.996	Not tolerated	1
953840	257A>G	Asp86Gly	Benign	0.197	Not tolerated	1
64425	259A>G	Ile87Val	Damaging	0.72	Tolerated	0
880997	274G>A	Glu92Lys	Damaging	1	Not tolerated	1
2154778	310C>T	Arg104Trp	Damaging	1	Not tolerated	1
880996	311G>A	Arg104Gln	Damaging	0.998	Not tolerated	1
962188	361C>T	Arg121Trp	Damaging	0.986	Not tolerated	0.79
880995	362G>A	Arg121Gln	Damaging	0.858	Tolerated	0.21
2175400	388C>T	Arg130Cys	Damaging	0.996	Not tolerated	1
2007326	389G>C	Arg130Pro	Damaging	0.921	Tolerated	0
2136689	389G>A	Arg130His	Benign	0.001	Tolerated	0
957488	395T>G	Ile132Ser	Damaging	1	Not tolerated	1
1517667	411C>A	Asp137Glu	Benign	0	Tolerated	0
2115626	419A>C	His140Pro	Damaging	0.941	Tolerated	0
2438523	446A>G	Asp149Gly	Benign	0.109	Tolerated	0
944289	460C>T	Arg154Trp	Damaging	0.997	Not tolerated	1
1006650	463C>A	Pro155Thr	Damaging	0.966	Tolerated	0
1991958	473G>A	Arg158His	Benign	0.388	Tolerated	0.11
1432897	519A>T	Arg173Ser	Benign	0	Tolerated	0.11
1971286	527C>T	Pro176Leu	Benign	0	Tolerated	0.84
1979236	541G>A	Asp181Asn	Benign	0	Tolerated	0.68
1036372	542A>G	Asp181Gly	Benign	0.087	Tolerated	0.68
1056222	565C>A	His189Asn	Benign	0	Tolerated	0.74
2521491	575C>G	Thr192Ser	Benign	0	Tolerated	0.63
1387284	610A>C	Asn204His	Damaging	0.703	Tolerated	0.21
2267460	613C>G	Leu205Val	Benign	0.263	Tolerated	0.21
1508246	634T>A	Ser212Thr	Benign	0.263	Tolerated	0.21
3064877	670C>T	Leu224Phe	Benign	0.011	Tolerated	0.21
2093377	683C>T	Pro228Leu	Damaging	0.976	Not tolerated	0.79
2119717	696C>G	Asp232Glu	Benign	0.059	Tolerated	0.11
2162565	720G>T	Lys240Asn	Damaging	0.81	Not tolerated	0.84
2179980	725T>C	Ile242Thr	Damaging	0.989	Not tolerated	0.84
1958573	759C>G	Asp253Glu	Benign	0	Tolerated	0.11
2074580	771G>C	Glu257Asp	Benign	0.008	Tolerated	0.11
1407164	775C>G	Gln259Glu	Damaging	1	Not tolerated	0.89
1516080	781G>A	Val261Ile	Benign	0.230	Tolerated	0.11
1162259	803T>C	Ile268Thr	Damaging	0.984	Not tolerated	0.89
3064965	820C>T	Arg274Cys	Damaging	1	Tolerated	0.05
7752	821G>T	Arg274Leu	Damaging	1	Tolerated	0.05
915348	821G>A	Arg274His	Damaging	0.998	Not tolerated	0.95
1504399	821G>C	Arg274Pro	Damaging	1	Tolerated	0.05
2067557	824C>T	Ser275Phe	Benign	0.271	Tolerated	0.05
2097936	845A>G	Asp282Gly	Benign	0.02	Tolerated	0
2506489	856T>C	Trp286Arg	Damaging	1	Not tolerated	1
1949259	869A>G	Asn290Ser	Benign	0	Tolerated	0.21
3339933	874G>C	Asp292His	Benign	0.003	Tolerated	0.05
1449972	886C>T	Arg296Cys	Benign	0.045	Tolerated	0.32
308882	889G>A	Val297Ile	Benign	0	Tolerated	0.11
2129305	901A>G	Thr301Ala	Benign	0	Tolerated	0
1338550	910G>A	Gly304Arg	Damaging	1	Not tolerated	1
7754	915C>G	His305Gln	Damaging	0.977	Not tolerated	1
7755	941T>G	Ile314Ser	Damaging	0.739	Not tolerated	1
308879	945G>T	Lys315Asn	Damaging	0.992	Not tolerated	1
2438524	967C>G	Leu323Val	Damaging	0.999	Not tolerated	1
7748	985G>A	Glu329Lys	Damaging	1	Not tolerated	1
2585094	985G>C	Glu329Gln	Damaging	1	Not tolerated	1
1118099	1015G>A	Val339Ile	Benign	0.001	Tolerated	0
860001	1016T>C	Val339Ala	Benign	0.345	Not tolerated	1
381603	1027C>T	Arg343Cys	Damaging	1	Not tolerated	1
2135373	1030C>T	Pro344Ser	Damaging	0.998	Not tolerated	1
7759	1036G>A	Val346Met	Damaging	0.998	Not tolerated	1
3188425	1040A>G	Gln347Arg	Benign	0.003	Tolerated	0
1037220	1045G>A	Ala349Thr	Benign	0	Tolerated	0
308878	1048G>A	Ala350Thr	Benign	0.001	Tolerated	0
727362	1073G>A	Arg358His	Benign	0.045	Not tolerated	1
754732	1085C>T	Thr362Ile	Benign	0.009	Tolerated	0
2429695	1088T>C	Leu363Pro	Damaging	1	Not tolerated	1
1038635	1102C>T	Arg368Cys	Damaging	1	Not tolerated	1
1342526	1103G>T	Arg368Leu	Benign	0.045	Tolerated	0
2519398	1108C>G	Arg370Gly	Damaging	0.957	Tolerated	0
840199	1109G>A	Arg370His	Benign	0,028	Tolerated	0
1040776	1115C>T	Pro372Leu	Benign	0.226	Not tolerated	0.89
882575	1121C>G	Pro374Arg	Damaging	1	Tolerated	0
1038600	1163C>A	Ala388Asp	Damaging	0.792	Not tolerated	1
7756	1171C>T	Arg391Cys	Damaging	1	Not tolerated	1
264696	1171C>A	Arg391Ser	Damaging	1	Not tolerated	1
2137319	1172G>A	Arg391His	Damaging	1	Not tolerated	1
882309	1183G>C	Glu395Gln	Benign	0.129	Tolerated	0
2747488	1186G>A	Glu396Lys	Damaging	0.995	Not tolerated	1
264697	1190A>C	His397Pro	Damaging	0.999	Not tolerated	1
2444136	1204C>T	Arg402Cys	Damaging	1	Not tolerated	0.63
2683191	1205G>A	Arg402His	Damaging	1	Not tolerated	0.63
2792281	1214C>T	Ser405Phe	Damaging	0.999	Not tolerated	0.63
1917095	1216T>A	Phe406Ile	Benign	0.026	Tolerated	0.37
992465	1229G>T	Cys410Phe	Benign	0.041	Tolerated	0.37
2099534	1273G>A	Glu425Lys	Damaging	0.981	Not tolerated	0.42

PolyPhen provides a probability score for how damaging a variant is, with higher values indicating more likelihood of pathogenicity, while SIFT predicts whether a substitution is “tolerated” or “not tolerated”, with a focus on protein functionality. The numbering of nucleotides is based on the DNA sequence of the *VDR* transcript NM_000376.3.

**Table 5:** Genetic variants found to be associated with vitamin D-related diseases in human studies.

Gene	Reference SNP Number	Molecular Consequences	Clinical Consequences
*CYP27B1*	rs778438734	Nonsense(Tyr7X)	Associated with an increased risk of vitamin D-dependent rickets type I (VDDR-I) due to the inhibition of active vitamin D synthesis [83].
*CYP27B1*	rs2140398340	Nonsense(Trp17X)	Associated with an increased risk of VDDR-I due to the inhibition of active vitamin D synthesis [84].
*CYP27B1*	rs760233049	Nonsense(Gln121X)	Associated with an increased risk of VDDR-I due to the inhibition of active vitamin D synthesis [85].
*CYP27B1*	rs2140397262	Nonsense(Gln135X)	Associated with an increased risk of VDDR-I due to the inhibition of active vitamin D synthesis [86].
*CYP27B1*	rs118204009	Missense(Arg389His)	Associated with an increased risk of VDDR-I due to the impaired 1-α-hydroxylase activity and reduced conversion of25-hydroxyvitamin D3 to its active form [87].
*CYP27B1*	rs118204011	Missense(Leu343Phe)	Associated with an increased risk of VDDR-I due to reduced 1-alpha-hydroxylase activity, leading to vitamin D deficiency [72].
*CYP27B1*	rs118204012	Missense(Glu189Gly)	A variant of uncertain significance reported to be associated with vitamin D insufficiency [73,88].
*CYP2R1*	rs1306247629	Nonsense(Tyr73X)	Associated with an increased risk of VDDR-1B due to disrupted CYP2R1 protein, resulting in impaired vitamin D activation [89].
*CYP2R1*	rs1555014321	Nonsense(Glu42X)	Associated with an increased risk of VDDR-1B due to truncated CYP2R1 protein, resulting in impaired vitamin D 25-hydroxylase activity [90].
*CYP2R1*	rs1848596931	Nonsense(Cys98X)	Associated with an increased risk of VDDR-1B due to truncated CYP2R1 protein, resulting in impaired vitamin D 25-hydroxylase activity [91].
*CYP2R1*	rs781823033	Nonsense(Arg131X)	Associated with an increased risk of VDDR-1B due to truncated CYP2R1 protein, leading to impaired vitamin D 25-hydroxylase activity and defective vitamin D metabolism [92].
*CYP2R1*	rs782006425	Nonsense(Trp234X)	Associated with an increased risk of VDDR-1B due to truncated CYP2R1 protein, resulting in impaired vitamin D 25-hydroxylase activity [92].
*CYP2R1*	rs199883994	Nonsense(Arg424X)	Associated with an increased risk of VDDR-1B due to truncated CYP2R1 protein, resulting in impaired vitamin D 25-hydroxylase activity [92].
*CYP2R1*	rs10741657	Promoter variant (Altered transcription)	Associated with decreased 25-hydroxyvitamin D levels and an increased risk of vitamin D deficiency, particularly in homozygous individuals [60].
*VDR*	rs121909792	Nonsense(Tyr295X)	Associated with hereditary vitamin D-resistant rickets (HVDRR), presenting with rickets, growth retardation, skeletal deformities, and alopecia [93].
*VDR*	rs1185429975	Nonsense(Ser187X)	Associated with HVDRR, presenting with rickets, hypocalcemia, and alopecia [59].
*VDR*	rs121909795	Nonsense(Gln152X)	Associated with HVDRR due to impaired binding of 1,25-dihydroxyvitamin D3 [94].
*VDR*	rs980041568	Nonsense(Arg73X)	Classified as pathogenic and observed in individuals with vitamin D-dependent rickets [95].
*VDR*	rs201106427	Nonsense(Arg50X)	Classified as pathogenic and observed in individuals with vitamin D-dependent rickets, presenting with alopecia and hypocalcemia [96].
*CYP24A1*	rs6068816	Silent (Arg159Arg)	Associated with an increased risk of hyperuricemia, particularly in overweight individuals [97].
*CYP24A1*	rs6022999	Intron variant	Associated with an increased risk of chronic hepatitis C virus infection due to disruptions in vitamin D metabolism [98].
*CYP24A1*	rs2762943	Promoter variant (Altered transcription)	Associated with low serum 1,25-dihydroxyvitamin D levels in multiple sclerosis patients [99].
*CYP24A1*	rs4809959	Intron variant	Increased risk of chronic systemic lupus erythematosus due to disruptions in vitamin D metabolism [100].
*CYP24A1*	rs17216707	Promoter variant (Altered transcription)	Associated with impaired vitamin D metabolism in kidney stone disease [101].
*CYP24A1*	rs6013905	Intron variant	Associated with impaired vitamin D metabolism in colorectal cancer patients [102].
*CYP24A1*	rs4809957	Intron variant	Associated with vitamin D deficiency in type II diabetes [103].
*CYP24A1*	rs17219315	Intron variant	Associated with altered vitamin D metabolism and autism in children [104].
*CYP24A1*	rs2296241	Silent (Pro289Pro)	Associated with an increased risk of hormone-related cancers [104].

All of the variants were selected from the literature and checked with the NCBI-supported public website PheGenI (The Phenotype–Genotype Integrator, ncbi.nlm.nih/gab/phegeni).

## Data Availability

Data are available with the corresponding author upon request.

## Abbreviations

25-OH Vit.D	25-Hydroxyvitamin D
7-DHC	7-Dehydrocholesterol
CYP	Cytochrome P450 Enzyme
GH	Growth Hormone
MS	Multiple Sclerosis
PTH	Parathyroid Hormone
RA	Rheumatoid Arthritis
RXR	Retinoid X Receptor
SNP	Single-Nucleotide Polymorphism
VDDR1	Vitamin D-Dependent Rickets Type 1
VDR	Vitamin D Receptor
VDRE	Vitamin D Response Element
Vit.D	Vitamin D
